# Internet-Based Medical Service Use and Eudaimonic Well-Being of Urban Older Adults: A Peer Support and Technology Acceptance Model

**DOI:** 10.3390/ijerph182212062

**Published:** 2021-11-17

**Authors:** Wenjia Li, Shengwei Shen, Jidong Yang, Qinghe Tang

**Affiliations:** 1 College of Communication and Art Design, University of Shanghai for Science and Technology, Shanghai 200093, China; liwenjia@usst.edu.cn; 2 School of Medicine, Tongji University, Shanghai 200092, China; 1933257@tongji.edu.cn; 3 School of Creativity and Art, Shanghai Tech University, Shanghai 201210, China; yangjd@shanghaitech.edu.cn

**Keywords:** urban elderly, internet-based medical service, eudaimonic well-being, peer support, technology acceptance model, aging design

## Abstract

Currently, internet services are developing rapidly, and the relationship between specific types of internet services and the well-being of older adults is still unclear. This study took a total of 353 urban older adults aged 60 years and above as research objects to explore the impact of the use behavior toward internet-based medical services (IBMS) on their well-being through an online questionnaire. This study integrated well-being theory and peer support theory, constructed an extended structural equation model of technology acceptance based on the technology acceptance model (TAM), and analyzed the variable path relationship. The results confirm the proposed model: older adults improved their eudaimonic well-being through using IBMS; perceived usefulness significantly affected the older adults’ attitudes towards IBMS; perceived ease of use significantly affected the use of IBMS through mediation; peer support significantly affected older adults’ attitudes, willingness, actual use, and well-being in the process. This study proposes that facilitating IBMS use for older adults in the development and design of internet technology programs should be considered in order to provide them with benefits. Moreover, paying attention to peer support among older adults plays an important role in the acceptance of new technologies and improving their well-being. The “peer support” of this study expanded and contributed to the research on the impact on older adults’ well-being and the construction of a technology acceptance model. The peer support in this study extended the influence factor of eudaimonic well-being and contributed to the further development of the TAM.

## 1. Introduction

China is now in an important period of implementing an active population aging strategy and building an intelligent society. In 2020, the number of older adults aged 60 and over in China reached 264 million, accounting for 18.7% of the total population. This number is expected to exceed 400 million in 2035, accounting for one quarter of the total population. According to statistics from the China Internet Development Report, the number of internet users in China reached 904 million in March 2020, and the rate of internet penetration reached 64.5%, but the proportion of internet users aged 60 and over was only 6.7%. As shown by data from the National Bureau of Statistics, the population aged 60 and above accounted for about 18.1% of the total population by the end of 2019 [1]. Following the emergence of internet technology, diverse media forms and applications have developed rapidly, changing the ways in which people communicate and live and also providing a new path of connection for people to pursue happiness. With the rapid development of digital and information technology, the internet health-care industry is developing at high speed. Internet medical use data suggest that using the Internet for medical treatment has become a common practice among many older people [2,3,4]. Therefore, the question focused on in the present study arises—that is, whether older adults’ use of the Internet for medical treatment will affect their happiness.

At present, research on the health and well-being of older adults mainly focuses on the fields of medicine, psychology, and sociology. However, there is little research on the relationship between internet medical care and the well-being of the elderly, which is closely connected to health management. Internet-based medical services (IBMS), taking the network as the carrier and information technology as the means, including mobile communication technology, cloud computing, the Internet of Things, big data, etc., have developed into a new type of medical and health service format by deeply integrating with traditional medical and health services. Their major functions include medical content, online diagnosis and treatment, online drug purchase, disease management, etc. Because of their rapid development and availability, IBMS have become one of the most important platforms for various medical and health institutions to provide health information, supports, and services to the demand side [5]. IBMS can benefit users in several aspects such as improving health knowledge and self-care skills and facilitating more appropriate medical decision making through online personalized medical consulting services. They can also improve the efficiency of medical care and reduce the burden of medical care, making sure that more medical tasks can be completed online and avoiding the traditional face-to-face medical pattern [6].

The health management needs of older adults mainly include health consultation, drug purchase, diagnosis and treatment of chronic diseases, etc. IBMS have potential benefits, which can greatly reduce the burden of time and energy required by older adults and their caregivers in many ways such as reducing transportation costs and effort, eliminating waiting times, making appointments accessible as well as making payment easy. However, their acceptance and utilization rate are still quite limited in older adults. The “digital barrier”, poor interactive experience, and technical operation difficulties experienced by older adults are often regarded as the reasons for their exclusion from all kinds of internet services. They often encounter difficulties in retrieving information and obtaining timely services from the Internet [7]. Notably, the popularity of internet technology and convenient network tools has increased over the last 20 years. During this process, the “new generation” of older people have preliminarily mastered some basic network skills through various auxiliary supports such as connecting to the Internet, searching for information, and downloading and installing mobile phone applications [8,9]. Especially with the growing popularity of smart phones, receiving information, entertainment, and other internet-based services have gradually become a part of the lives of the elderly. The contradiction above suggests that the mechanism behind the use of IBMS by older adults is not yet fully understood and is worthy of further exploration.

Studies into population health technology acceptance have primarily used the technology acceptance model (TAM) or its updated versions as a central theoretical framework [10,11]. Although the classical TAM has wide applicability, it still needs to be modified by additional contextual and theoretically reasonable factors, which can improve the prediction ability of the model in a specific context [12]. External support is often regarded as an important factor for older adults to accept new technologies. Previous studies on support for older adults to participate in internet services mainly focused on the study of “digital feedback” from their children and other family members, while a growing number of studies show that peer groups play an important role in the behavior decision making of older adults [13,14,15,16]. The older adults peer group is a spontaneous population, and the members of the group are roughly of the same age and social status. They have common life experience, values, and family background. More importantly, age factors cause this group to show a certain convergence in health concepts and health management. In this homogeneous group, members interact according to the default rules. The exchange is not only the operation technology but also the group understanding of health concepts and health management. Thus far, there are few reports on the role of peer support in the elderly’s participation in IBMS and its impact on the elderly’s well-being.

Therefore, this study attempted to explore the relationship between internet medical participation and the well-being of urban older adults by constructing an extended TAM with the addition of the influencing factor of “peer support”. The novelty of this study is that it aimed to test an extended version of the TAM. This version combines the availability of IBMS and peer support theory to detect the acceptance of IBMS by older people, discusses the impact mechanism of the elderly’s realization of well-being through specific types of internet use, and explores the impact of IBMS on the well-being of older adults so as to put forward suggestions to improve the happiness of older adults from the perspective of internet health and peer support.

## 2. Literature Review and Hypotheses

### 2.1. Internet Use and the Pursuit of Well-Being by Older Adults

Well-being is a widely studied topic in psychology and sociology [17,18,19]. It is largely accepted that subjective well-being (SWB) is defined as the overall evaluation of an individual’s quality of life according to self-determined standards, and it is often measured by three dimensions (i.e., life satisfaction, positive emotion, and negative emotion) [20]. Psychological well-being is defined as a good psychological state including six dimensions: self-acceptance, positive relations with others, autonomy, environmental mastery, life goals, and personal growth [21]. Based on the theory of self-actualization, self-determinism, and self-teleology, Waterman integrated subjective well-being and psychological well-being and constructed a contemporary concept of eudaimonic well-being (EWB), referring to the degree to which individuals identify, develop, and use their own potential to improve their life significance [22,23]. The Questionnaire for Eudaimonic Well-Being (QEWB) was developed to measure the extent of individual well-being. EWB includes several autonomy elements such as life satisfaction, life goals, and personal growth [24,25]. Moreover, experiences of action and self-realization in society are also valued. EWB emphasizes the sense of purpose and meaning that an individual feels in life. It is not only the subjective experience of an individual’s emotions or psychological feeling but also the fulfillment of targeted activities and behaviors when facing new opportunities and challenges. Thereby, the individual can achieve self-satisfaction from it, and this self-satisfaction is more important for personal development than well-being [26]. This new definition of well-being has great significance for the life quality of older people, along with their physical and psychological health as well as social harmony.

The relationship between internet use among older adults and their happiness, as well as its influential factors, has been studied previously [2,27]. Multiple studies have found that internet use has a significant incentive effect on older adults by promoting their self-improvement and thereby affecting their life satisfaction and well-being [2,28]. Older person have various motivations for using the Internet, which can be divided into three types including practical (acquiring useful skills for life, gathering data, and integrating oneself into the information society), developmental (following technological progress, acknowledgement and enhancement of self-respect), and social (meeting peers or contacting relatives and friends) [29]. Important motivations for older adults to use the Internet are based on needs such as maintaining social connections, exchanging knowledge or life experience, and bridging the generation gap [30].

IBMS are a special type of internet-based service, from which older adults can not only obtain health guidance but also communicate with each other. Compared with other forms of internet-based services, IBMS are relatively new, with technological content for the elderly. On the one hand, when older adults access these applications, internet medical services can: (i) solve practical problems related to health management and reduce the burden on caregivers; (ii) enhance their learning ability and confidence [8,31]; (iii) alleviate their social alienation and anxiety, while improving their self-efficacy, sense of control, and life satisfaction. On the other hand, as with other internet-based services, older people often lack confidence in their learning ability and even refuse to accept new knowledge, and the main reasons for this include a lack of technical support and poor understanding about the parameters, functions, and payment.

These studies suggested that, as a new medical model, IBMS are both difficult and convenient in health management for older adults. Whether they can be accepted and used among them is regarded as a goal behavior of personal growth and self-affirmation and also meets the basic essentials of EWB. Therefore, the concept of EWB was adopted in this study.

### 2.2. Effect of Peer Support on Older Adults

Peer group refers to a spontaneous informal group of individuals that share similar characteristics (such as age, interests, values, and background) with each other [32,33]. Zhao [34] divided peer groups into four types: distance proximity type, experience similarity type, group activity type, and congenial type. It was found that peer groups had similar characteristics, and it was easier to understand the problems they faced than other social networks. In terms of function, peer groups have a significant role in the development of individual behavior and has certain positive effects on individuals such as guidance, constraint, cohesion, motivation, and adjustment. Peer support includes information support, emotional support, and other support obtained from peer groups with similar experiences or whose members are under the same pressure or situation so as to help solve existing or potential problems [15,16].

At present, studies on peer support related to older adults mostly focus on peer-to-peer assistance among elderly patients [35,36]. This “support” mainly refers to the mental and physical help obtained by the elderly during interactions. These studies show that peer assistance can alleviate the anxiety and depression of the elderly with chronic diseases effectively, enhance disease cognition, reduce social isolation and loneliness in older adults through information exchange, and directly affect their health outcomes or behavior. Older adults’ peer support can also help them to integrate into society and build a social support network effectively. Research by Jiang showed that peer support helps older adults accept new things. This study on the usage of smart phones among the elderly showed that in the face of the digital divide, older adults have begun to embark on a spontaneous exploration of these technologies in their daily lives [37]. Since their children or grandchildren do not always have the time and patience to give them enough feedback and help, Chinese elderly people often seek support from peer groups who are experiencing the same difficulties in order to solve information technology problems. When people who support or are being supported have similar problems and experiences, they are more likely to generate a sense of belonging and security and find it easier to accept each other’s support.

In the research on the well-being of older adults, social support, health status, intergenerational support, and pension services are considered to be the determinants [33,38,39]. Peer support, as an essential part of individual social support, can provide external support for individuals, and it exerts many functions such as emotional communication, resolution of psychological conflict, solutions to daily confusion, and improvement in the learning of skills [40]. Although little research has explored the relationship between peer support and the well-being of older adults, the above-mentioned benefits of peer support led us to believe that it has a positive effect on the well-being of the elderly.

### 2.3. Technology Acceptance Model (TAM) 

The classical technology acceptance model (TAM) has six dimensions/constructs including perceived ease of use, perceived usefulness, external variables, use attitude, behavior intention, and actual use behavior [41,42]. The TAM is considered to be one of the most influential models for explaining the behavior of technology use, and it is commonly applied in the acceptance research of new technologies, new ideas, and new things as the most widely used theoretical model [43]. Based on the classical TAM, this study aimed to construct an extended theoretical model.

#### 2.3.1. Perceived Usefulness and Perceived Ease of Use

Perceived usefulness is defined as the extent to which an individual supposes that using technology will improve task performance, and perceived ease of use refers to the extent to which an individual thinks that using technology will require no effort. In view of internet use, both perceived usefulness and perceived ease of use have certain effects on users’ attitudes and behavioral intentions [44]. Previous studies indicated that perceived usefulness is an influential factor in older people’s participation in the Internet [9,45]. For older adults, as a new technological tool, the functions of internet medical services are far more complicated than previous simple services such as online appointments. Will the usefulness and ease of use directly affect their attitude and willingness to use these services and even their behavior? According to the above description, this study puts forward the following research hypotheses:

**Hypothesis** **1** **(H1).** *Perceived usefulness is positively correlated with the attitude of urban older adults to participate in IBMS.*

**Hypothesis** **2** **(H2).** *Perceived ease of use is positively correlated with the attitude of urban older adults to participate in IBMS.*

**Hypothesis** **3** **(H3).** *Perceived ease of use is positively correlated with perceived usefulness.*

#### 2.3.2. Attitude toward Use, Behavioral Intention, and Actual Use

Attitude toward use refers to a person’s positive or negative feelings when using the system, as well as evaluating the impact of performing certain behaviors. Behavioral intention is used to measure the intensity of a person’s willingness to perform a specific behavior. Zeng’s study pointed out the relationship between attitude toward use and behavioral intention, namely, that attitude directly affects behavior [46]. A study on a TAM regarding mobile reading confirmed that attitude toward use has a significant positive impact on behavioral intention. Intention to use is a type of behavioral intention [47], and people’s attitudes towards a certain substance or behavior will affect the generation of people’s behavioral intention and ultimately determine the implementation of behavior. The stronger the behavior intention, the easier it is to put it into action [48,49]. Therefore, the following hypotheses were proposed:

**Hypothesis** **4** **(H4).** *The attitude of urban older adults to participate in IBMS is positively correlated with their behavioral intention.*

**Hypothesis** **5** **(H5).** *The behavioral intention of urban older adults to participate in IBMS is positively correlated with their actual use.*

#### 2.3.3. Peer Support

According to the above literature review in Section 2.2, peer support will probably affect the well-being of older adults positively. Meanwhile, previous studies regarded digital feedback or intergenerational assistance as an important external variable for older adults to accept internet services [14,50,51]. Similarly, this study took peer support as an important independent variable and investigated whether psychological and technical guidance from peers can affect their behaviors and well-being in the process of their participation in IBMS. This study puts forward the following hypotheses:

**Hypothesis** **6a** **(H6a).** *Peer support is positively correlated with the attitude of urban older adults to use IBMS.*

**Hypothesis** **6b** **(H6b).** *Peer support is positively correlated with the intention of urban older adults to use IBMS.*

**Hypothesis** **6c** **(H6c).** *Peer support is positively correlated with actual use of IBMS by the urban older adults.*

**Hypothesis** **6d** **(H6d).** *Peer support is positively correlated with the EWB of the urban older adults.*

#### 2.3.4. Eudaimonic Well-Being

As previously mentioned [23], on the basis of the definition of EWB, the QEWB was developed to measure the extent of individual happiness. As an emerging technological tool, user experience with internet medical services is a key factor in the process of self-development and self-confirmation in urban older adults, which is closely related to their EWB. Therefore, this study assumes that:

**Hypothesis** **7** **(H7).** *The participation of urban older adults in IBMS is positively correlated with their realization of well-being.*

**Figure 1:**
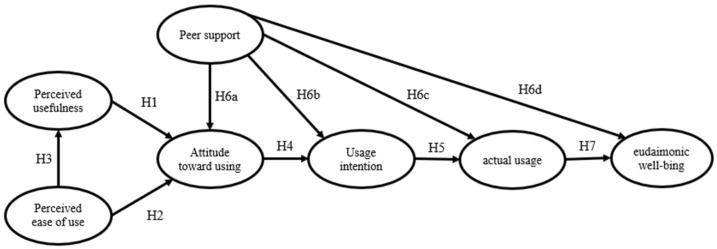
The proposed research model.

Based on the above theoretical assumptions and the TAM model, this study constructed a structural equation model of internet use and EWB among urban older adults (Figure 1).

## 3. Materials and Methods

### 3.1. Research Objects and Data Collection

Data collection was conducted through an online self-reported survey supported by the Sojump website, a widely used questionnaire website on the Chinese mainland. The research subjects in Shanghai and the neighboring Yangtze River Delta region were recruited, and questionnaires were distributed through WeChat chat groups, a popular virtual community in China. It was required that those who filled out the questionnaires were 60 years old or above and knew about or had used internet medical-based services. A total of 400 questionnaires were distributed. Excluding invalid questionnaires (quiz time < 2 min) and repeated answers, the remaining 353 valid questionnaires were used in the following research. After summarizing several studies, Rappaport et al. [52] considered that a sample size of 100–150 was the minimum requirement for structural equation model analysis. Therefore, the sample size met the requirements of this study.

### 3.2. Research Variables and Measurements

#### 3.2.1. Operationalization of Research Variables

The concept (variable) of the present research model was defined according to the research topic on the basis of the existing relevant research concepts, and then the observation indexes or items were set. The operational definitions and sources of the eight constructs are shown in Table 1.

#### 3.2.2. Measurement and Hypothesis

On the basis of fully drawing lessons from previous studies and meeting the requirements of at least three items per construct in the structural equation model, each construct had three or more measurement items in this study [54]. The cognitive interview is a widely used method to identify sources of confusion in questionnaires by focusing on the cognitive process of respondents answering the questionnaire items [55]. We also consulted two questionnaire design experts to guarantee the quality of the questionnaire. All test structures were measured using a 7 point Likert scale, ranging from “strongly disagree” to “strongly agree”, namely, 1 for “strongly disagree”, 2 for “disagree”, 3 for “slightly disagree”, 4 for “not sure”, 5 for “slightly agree”, 6 for “agree”, and 7 for “strongly agree”. The objective availability was measured by task performance, namely, the number of tasks successfully completed per unit of time (minute).

Perceived usefulness included three items: (1) IBMS are an indispensable tool for me in the process of seeking medical advice; (2) the medical services provided by IBMS could meet my needs; (3) IBMS make it more convenient for me to communicate with doctors and other patients.

Perceived ease of use included three items: (1) I do not think the operation of IBMS is complicated; (2) if I encounter operational problems, I can easily seek help to solve them; (3) I can use various functions provided by IBMS.

Attitude toward use included four items: (1) I think using IBMS is wise; (2) I think using IBMS is pleasant; (3) I think IBMS can do more things; (4) I will continue to use IBMS.

Behavioral intention included three items: (1) I am willing to recommend IBMS to my relatives and friends; (2) I share my experience of using IBMS so that more people can conveniently use them; (3) I am willing to learn to use IBMS related to new functions.

Peer support included three items: (1) my peers encourage me to use IBMS; (2) I master some functions of IBMS through peer learning; (3) when I encounter problems in the process of using IBMS, I can seek solutions from peers.

Actual use included four items: (1) I always use IBMS in the process of seeking medical advice; (2) I can acquire the needed information from IBMS and communicate with people; (3) I am familiar with the payment process of IBMS; (4) I have been using IBMS for more than 3 years.

EWB, which refers to “self-discovery”, “sense of purpose and significance of life” in the QEWB as well as “cognitive evaluation of life quality” in SWB, included five items: (1) learning to use IBMS makes me feel confident; (2) I am satisfied with being able to learn new medical information through IBMS; (3) looking back on the past, I feel the meaning of life; (4) I feel that my current living condition is good; (5) most aspects of my life meet my ideals [20,21,24].

#### 3.2.3. Data Analysis

The internal consistency of the construct was assessed by Cronbach’s α reliability (acceptable if >0.7) and structural reliability (acceptable if >0.6) [56,57,58]. The convergence and discriminant validity of the measurement model were evaluated by confirmatory factor analysis. If all terms’ factor loads were significant and greater than 0.50, the convergence effectiveness was verified. Structural equation models were used to test hypothetical models. The eight most widely used fitting indexes in SSCI studies were used to evaluate the overall model fit. The ratio between chi-square statistics and the degree of freedom (χ^2^/df < 3), the χ^2^ chi-square, the approximate root mean square error (RMSEA), the normed fit index (NFI), the Tucker–Lewis index (TLI), the comparative fit index (CFI), the goodness of fit index (GFI), and the adjusted goodness of fit index (AGFI) were compared. The evaluation and analysis of the SEM in the present study was completed with AMOS24.0 (IBM Corporation Armonk, NY, USA) and SPSS 26.0 (IBM Corporation Armonk, NY, USA).

## 4. Results

### 4.1. Sample Characteristics

The demographic data of the study sample (*n* = 353) were as follows: gender, 149 males (42.21%) and 204 females (57.79%); age, 96 subjects (27.20%) were aged between 61 and 65, 97 subjects (27.48%) were aged between 66 and 70, 77 subjects (21.81%) were aged between 71 and 75, 62 subjects (17.56%) were aged between 76 and 80, and 21 subjects (5.95%) were aged over 81; educational background, 93 subjects (26.35%) were educated below a junior high school level, 109 subjects (30.88%) were educated at a junior high school level, and 151 subjects (42.78%) were educated at or above university level; residence status, 166 (47.03%) subjects lived with their spouses, 107 (35.971%) subjects lived with their families, and 60 (17.00%) subjects lived alone. The gender ratio of this sample was also in line with the normal ratio of the current older population. As the average life expectancy of China’s female population is higher than that of men, the aging degree of the female population is also higher than that of men. In the last four decades, female older populations over 65 and over 80 have consistently been greater than the male older populations in these age brackets with a comprehensive ratio of 58.19:41.81 [40]. The sex ratio of this study sample was also in line with the current normal proportion of the older population.

### 4.2. The Reliability, Validity, and Discriminant Validity of the Measurement Model

The factor loads of this study ranged from 0.725 to 0.878, indicating that each item had reliability. The Cronbach’s α coefficient of all constructs ranged from 0.798 to 0.915, and the composite reliability (CR) ranged from 0.782 to 0.907, indicating that each construct had good internal consistency. Average variance extraction (AVE) ranged from 0.545 to 0.738 (Table 2) in accordance with the standards. Therefore, all seven dimensions had good reliability and convergent validity. A rigorous AVE method was used to test the discriminant validity of the measurement model in our research. If the AVE square root of each construct was larger than the correlation coefficient among the constructs, the model had discriminant validity. As shown in Table 3, the AVE root mean square of diagonal constructs in this study was larger than the correlation coefficient outside the diagonal lines. Accordingly, most constructs in this study had good discriminant validity.

### 4.3. Fitting Degree of the Structural Model

The eight most widely used fitting indexes in SSCI studies are as follows: the model fitting degree of χ^2^ chi-square was 509.6; *p* < 0.001; the allowable range of normed chi-square (χ^2^/DF) was 1 < χ^2^/DF < 3; the model fitting degree was 1.53. The allowable range of RMSEA was below 0.08, and the model fitting degree was 0.069. The allowable range of NFI was above 0.9, and the model fitting degree was 0.91. The allowable range of TLI (NNFI) was above 0.9, and the model fitting degree was 0.97. The allowable range of CFI was above 0.9, and the model fitting degree was 0.96. The allowable range of GFI was larger than 0.9, and the model fitting degree was 0.92. The allowable range of AGFI was higher than 0.8, and the model fitting degree was 0.91. According to the allowable range, each fitting degree of this model met the requirements.

### 4.4. The Structural Model Path Analysis and Test Results

**Figure 2:**
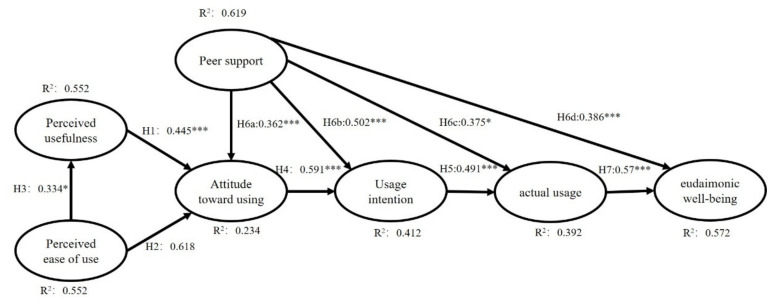
The final model with parameter estimates of the significant paths. * *p*-value < 0.05, ** *p*-value < 0.01, and *** *p*-value < 0.001.

Figure 2 shows the results of the estimated structural model, and Table 4 summarizes the results of the hypothesis testing. The statistical data in this study basically support the hypothesis of the model. From the structural path in Figure 2 and the hypothesis testing in Table 4, it is indicated that the other seven hypotheses were all supported by attitude towards use, except for perceived ease of use.

#### 4.4.1. Significant Influence among Variables in the Structural Model

Table 4 shows the significant results of the influence among path coefficient variables: perceived usefulness (PU) (β = 0.445, *p* < 0.001) and peer support (PS) (β = 0.362, *p* < 0.001) had significant effects on attitude toward using (ATU). ATU (β = 0.591, *p* < 0.001) and peer support (PS) (β = 0.502, *p* < 0.001) significantly affected behavioral intention. Peer support (β = 0.375, *p* = 0.018) and behavioral intention (BI) (β = 0.490, *p* < 0.001) significantly affected actual use (AU). Peer support (β = 0.586, *p* < 0.001) and AU (β = 0.570, *p* < 0.001) significantly affected EWB. Perceived ease of use (PEU) (β = 0.334, *p* = 0.021) significantly affected PU, with no significant direct impact on attitude (β = 0.168, *p* = 0.092), but PEU had a significant indirect influence on ATU through perceived usefulness. Perceived usefulness not only mediated PEU and ATT, but also had a significant direct impact on ATT.

#### 4.4.2. Peer Technological Support Exerted Significant Influences on Attitude toward Use, Behavioral Intention, Actual Usage, and Eudaimonic Well-Being

According to the results of non-standardized coefficients (Table 4), PS had significant effects on ATU (β = 0.362, *p* < 0.001), BI (β = 0.502, *p* < 0.001), AU (β = 0.375, *p* = 0.018), and EWB (β = 0.586, *p* < 0.001).

#### 4.4.3. Explanatory Power of Older Adults’ Perception and Actual Use of IBMS to Eudaimonic Well-Being

The explicable variance data showed that this model had a good explanatory power for older adults participating in IBMS and EWB. The explanatory power of PEU to PU was 55.2%; the explanatory power of PEU, PU, and PS to ATU was 23.4%. The explanatory power of ATU and PS to BI was 41.2%. The explanatory power of PS and BI to AU was 39.2%; the explanatory power of PS and AU to EWB was 57.2%.

## 5. Discussion

This study exhibited several differences from the traditional TAM: firstly, by adding “peer support” as an external variable, we examined whether it had an impact on the path from attitude to usage intention and then to actual usage in the course of older adults using IBMS, and we also confirmed whether it could eventually affect well-being instead of affecting PU and PEU. Secondly, we took actual usage in the original model as an independent variable and regarded EWB as a dependent variable. Our study constructed an extended structural equation model of use behavior and EWB, and discovered the relationship between peer support, internet usage, and EWB through the test results of this model. Following the application of peer support theory, we explored the internal mechanism of this relationship and also expanded the research on the use of IBMS and EWB to some extent.

The hypothesis that “peer support” has a significant impact on the attitude, usage intention, actual use, and EWB of older adults in participating in IBMS has been well supported. There were three significant factors to consider when analyzing the observed variable of peer support: firstly, in terms of technology use, this study was similar to many studies related to “peer assistance” and proved its promoting role in behavioral decision making in the older adults [15,16]. As important sources of social support for older adults, peer groups, such as friends, colleagues, and neighbors, are often used as an alternative to the “intergenerational support” of their children. Older people often show a certain convergence in health concepts and management. Compared with their children, they are faced with similar technological difficulties and learning experiences. The accumulation of experience in the process allows members to share experiences, learn skills, update concepts, and help older adults to solve general operational problems through benign interaction. Different from the so-called “digital feedback”, the support of peer groups solves more specific problems that older adults encounter in IBMS and also provide informational and emotional support to each other by means of geographical and psychological proximity, which can encourage older adults to continue to use IBMS. Secondly, the influence of peer support on the EWB of older adults in the past is mostly considered to be related to financial support, emotional support, and daily care [8]. However, this study confirmed that technical and psychological support provided by peers could affect the older adults’ use of IBMS from various points of view, including attitude, usage intention, and actual use, and also found that peer support played a key role in the older adults’ achievement and promotion of EWB. IBMS could reduce part of the burden of older adults in the process of medical treatment [7]. For the urban older adults, IMBS are also an effective supplement to the existing social networking services. The older people in this study were gradually reducing their social activities because of their own or their partner’s disease. In addition, there are also some older people who migrate with their children, becoming “older adult drifters”. In the new environment, they take on an inactive part in social interactions, which causes them to have a weak social communication network and a poor understanding of the community. As a crucial part of peer support, a good social support network can not only enrich older adults’ lives and enhance social support but also increase their sense of belonging and security. Thirdly, at the level of the theoretical model, although some studies have examined the influence of the digital generation gap and digital barriers on older adults and explored the role of “digital feedback”, no studies have yet taken peer support as a measurement index for further investigation [13]. This study operationalized peer technical and psychological support to test whether and how this “independent variable” affected the participation of urban older adults in the process of using IBMS, which was proven by data through assumptions. This concept could be an important predictor for the promoting effect of peer support on older people’s attitudes, intentions, and AU of IBMS.

The aforementioned research on internet use and EWB among older adults proved that internet use had a certain positive impact on the physical and psychological healing of the older adults and had a certain effect on their self-cognition, social interaction, life satisfaction, and self-confidence [2,59,60]. Our study also confirmed the positive effects of IBMS use on the life satisfaction and self-confidence of older adults. However, the difference was that our research took the participation of urban older adults in IBMS as an independent variable and regarded IBMS use as one of the daily technical use activities and life goals rather than internet use in general. By observing the self-confirmation of older adults during their information technology activities and whether they have achieved happiness or not, the influence of mobile internet use on EWB needs to be further discussed.

Existing studies on older adults’ well-being mainly focus on their living conditions, physical and mental health as well as the external pension system, or they focus on subjective well-being based on emotion. No studies have paid attention to older adults’ participation in IBMS, access to health support services, and their impact on self-health assessment and the activeness of life. The main emphases of our research included the activeness of older people’s lives and their behavior in the information age, and the subjectivity of their behavior and self-efficacy. The three types of motivation for older adults to learn to use information technology included acquiring useful healthy management skills by means of practical motivation (such as online drug purchases and online appointments), paying attention to various services through the Internet (such as seeking online guidance, diagnosis, and treatment), or communication with social relatives, friends, and peers (such as sharing medical experiences and updating health concepts) [31]. No matter the older person’s motive, our study suggests that older adults have gradually accepted the practicability and usefulness of the new program subjectively. As an alternative to traditional walk-in medical services, choosing IBMS will enable older adults to spend more time and energy solving the problems they encounter. This process includes the determination of life goals and activities to achieve these goals and meets the basic essentials of EWB.

There are three differences between this study and existing research on the EWB of older adults and its influencing factors. Firstly, older adults’ EWB is a more active perception and experience regarding the self-realization of a meaningful life, which enriches the existing happiness theory for older adults. Secondly, with the background of information technology development, the proposal that peer support affects the EWB of older adults reflects the mutual assistance in the field of health among the elderly, which is an extension of the theory of influencing factors of older adults’ EWB; thirdly, this study constructed a model regarding older adults’ participation in IBMS and their EWB, adding the independent variable of PS and the dependent variable of EWB to extend the TAM. This will deepen and extend the research on internet use and EWB in the older adults and may become the starting point of future research on the relationship between internet technology acceptance and well-being.

Not surprisingly, our study found that PU directly affected ATU. If the older person formed a positive attitude toward IBMS, they would be more willing to use this service. This result shows that older people were more concerned about the usefulness of IBMS in their health care and whether it could solve practical health-related issues. Peer technical support directly affected older people’s attitudes, willingness, and actual usage of IBMS, indicating the importance of variables. In previous study, Wong et al. found that PEU rather than PU was an important predictor for older people’s behavioral intention to use online health information [60]. In contrast, our study showed that PEU did not have a direct impact but instead had an indirect influence through PU. This result confirms the relevant research on the relationship between internet anxiety and PEU to some extent, since internet anxiety will make users realize that the technology is difficult to operate and reduce the evaluation of the usability of internet medical systems, which eventually affects BI. The results of this study also imply that improving older adults’ awareness of using IBMS is an urgent problem that needs to be solved. The fast-developing information technology serves humanity and should avoid causing feelings of exclusion among the older adults. In order to help older adults adapt to the development of information technology, intelligent medical program development should consider how to benefit older adults and provide more simple and easy-to-operate programs for them. Digital technology training for older adults is also necessary to help them cross the digital divide so that IBMS can become a good helper in the daily lives of older adults, improve their quality of life and, thus, promote active aging [61].

We recognize some limitations in our study. First, limited by manpower and time, our research was conducted in the Yangtze River Delta region, where both the internet penetration rate and education level of older persons are higher. These results may not be applicable to other older adults, and it is suggested that future studies should include participants from different regions to solve this limitation. Secondly, the self-assessment method used to measure the EWB of older adults has certain limitations. Interviewees will have inconsistent understandings in the process of perceiving facts and self-answering questionnaires. In the follow-up study, questions and scales should be more accurate to avoid ambiguity in understanding [62,63,64]. Finally, as with most previous studies, our study only adopted a cross-sectional survey. Some people believe that personal views on technology may change over time. Therefore, the current results can be used as a baseline for future longitudinal studies so as to check for possible changes in older persons using IBMS [65]. Furthermore, the mediating role of variables, especially the mediating role of peer support in this model, is worthy of further exploration. This research will continue in future work.

## 6. Conclusions

With the increasing popularity of internet medical care, subjectively, older people gradually accepted its practicability and usefulness. Reducing the technical operating threshold through various measures to benefit older adults will help to improve the utilization rate of internet medical services and improve the happiness of older adults. The needs of older adults in health management and internet medical care are multi-level including diagnosis and treatment needs, information updates, technical support, social communication, and psychological counseling. The present research shows that peer support contributed to improving the acceptance of new technologies, the quality of life and the well-being of the older adults. These findings provide important enlightenment for the design and implementation of older adult care strategies and expand and contribute to the research on the impact on older adults’ well-being and the construction of technology acceptance models.

## Figures and Tables

**Table 1:** The operationalization and sources of the questionnaire items.

Variables	Operationalization	Source
Perceived Usefulness (PU)	To what extent do urban older adults think the application of IBMS is useful	Xu, Y. et al. (2020) [2] Amini, R. et al. (2019) [3]
Perceived Ease of Use (PEU)	To what degree do urban older adults consider IBMS as being easy to use	Jun, W. (2020) [7] Banerjee, S. (2018) [26] Disler, R.T. et al. (2019) [5]
Attitude Toward Using (ATU)	Evaluating the attitude of urban older adults about using IBMS	Amini, R. et al. (2019) [3] Wong, C.K. et al. (2014) [53]
Behavioral Intention to Use (BI)	The intention explaining why users recommend, share, or continue to use IBMS now or in the future	Huang, S. et al. (2015) [47]
Actual Using (AU)	The usage of IBMS by urban older adults	Sun, X. et al. (2020) [8] He, J. et al. (2020) [14]
Peer Support (PS)	Whether peers support the use of IBMS	Ong, B.N. (2020) [50] Legg, M. et al. (2012) [36]
Eudaimonic Well-Being (EWB)	How older adults feel about their personal meaning and goals in life	Waterman, A.S. et al. (2010) [24] Straume, L.V. et al. (2012) [26]

**Table 2:** Reliability and validity of the measurement model in this research.

	Item	Factor Load	Cronbach’s α	CR	AVE
PU	PU1	0.742	0.806	0.807	0.582
	PU2	0.781			
	PU3	0.765			
PEU	PE1	0.834	0.867	0.894	0.738
	PE2	0.878			
	PE3	0.864			
PS	PS1	0.794	0.811	0.812	0.590
	PS2	0.751			
	PS3	0.758			
ATU	ATU1	0.828	0.915	0.907	0.709
	ATU2	0.838			
	ATU3	0.874			
	ATU4	0.826			
BI	BI1	0.729	0.798	0.782	0.545
	BI2	0.761			
	BI3	0.725			
AU	AU1	0.862	0.905	0.905	0.704
	AU2	0.852			
	AU3	0.819			
	AU4	0.823			
EWB	EWB1	0.735	0.874	0.870	0.573
	EWB2	0.766			
	EWB3	0.768			
	EWB4	0.732			
	EWB5	0.783			

PU: perceived usefulness; PEU: perceived ease of use; PS: peer support; ATU: attitude toward using; BI: behavioral intention to use; AU: actual using; EWB: eudaimonic well-being.

**Table 3:** Discriminant validity of the measurement model.

	AVE	PS	PEU	PU	ATU	BI	AU	EWB
PS	0.59	0.768						
PEU	0.738	0.632	0.859					
PU	0.582	0.531	0.721	0.763				
ATU	0.709	0.682	0.703	0.601	0.842			
BI	0.545	0.674	0.342	0.421	0.712	0.738		
AU	0.704	0.514	0.240	0.384	0.623	0.652	0.839	
EWB	0.573	0.498	0.238	0.365	0.603	0.587	0.654	0.757

PU: perceived usefulness; PEU: perceived ease of use; PS: peer support; ATU: attitude toward using; BI: behavioral intention to use; AU: actual using; EWB: eudaimonic well-being.

**Table 4:** Path analysis and detection results of the structural model.

Hypotheses	Structure Pattern Path	Path Coefficients	*p*-Value	Supported?
H1	PU→ATU	0.445 ***	0.000	Support
H2	PEU→ATU	0.168	0.092	Not support
H3	PEU→PU	0.334 *	0.021	Support
H4	ATU→BI	0.591 ***	0.000	Support
H5	BI→AU	0.490 ***	0.000	Support
H6a	PS→ATU	0.362 ***	0.000	Support
H6b	PS→BI	0.502 ***	0.000	Support
H6c	PS→AU	0.375 *	0.018	Support
H6d	PS→EWB	0.586 ***	0.000	Support
H7	AU→EWB	0.570 ***	0.000	Support

PU: perceived usefulness; PEU: perceived ease of use; PS: peer support; ATU: attitude toward using; BI: behavioral intention to use; AU: actual using; EWB: eudaimonic well-being. * *p*-value < 0.05, and *** *p*-value < 0.001.

## Data Availability

Upon reasonable request, data used and analyzed during the current study are available from the corresponding author.
